# Surrogates 20 years on: long-term psychological health, contact with surrogacy families, and thoughts and feelings about post-birth contact

**DOI:** 10.1093/humrep/deaf234

**Published:** 2025-12-12

**Authors:** V Jadva, K Shaw, P Hall, S Ross, S Imrie

**Affiliations:** Department of Psychology and Neuroscience, City St Georges, University of London, London, UK; Centre for Family Research, University of Cambridge, Cambridge, UK; EGA Institute for Women’s Health, University College London, London, UK; Centre for Family Research, University of Cambridge, Cambridge, UK; Centre for Family Research, University of Cambridge, Cambridge, UK; EGA Institute for Women’s Health, University College London, London, UK; Centre for Family Research, University of Cambridge, Cambridge, UK

**Keywords:** surrogacy, gestational, traditional, surrogate, follow-up, psychological health, mental health

## Abstract

**STUDY QUESTION:**

What were the psychological health outcomes for surrogates 20 years after the surrogacy pregnancy?

**SUMMARY ANSWER:**

Most surrogates did not experience psychological problems 20 years after the pregnancy with many showing positive psychological wellbeing.

**WHAT IS KNOWN ALREADY:**

Studies of surrogates from the global north have found that although some surrogates may experience psychological difficulties in the weeks following the birth of the child, this decreases over time, with most surrogates not experiencing psychological problems at 6 months, 1 year or 10 years following the birth.

**STUDY DESIGN, SIZE, DURATION:**

Cross-sectional follow-up study of 21 surrogates who had conducted surrogacy ∼20 years previously (*M *= 20.33 years, *SD *= 3.31, range 13–26 years). Data are presented from phase 3 of the study. All participants were interviewed and 17 completed psychometric scales. Data were collected between December 2021 and September 2022.

**PARTICIPANTS/MATERIALS, SETTING, METHODS:**

Twenty-one surrogates participated in the study. Ten surrogates (48%) had completed only gestational surrogacy arrangements, five (24%) had completed only traditional surrogacy arrangements, and six (29%) had completed both traditional and gestational surrogacy arrangements. All were domestic arrangements for heterosexual couples. Data were collected using semi-structured interviews and standardized psychometric questionnaires to assess experiences of surrogacy and psychological health of surrogates. Data on frequency of contact and relationship with the surrogacy family were also obtained.

**MAIN RESULTS AND THE ROLE OF CHANCE:**

Seventeen of the 21 surrogates completed the questionnaires assessing mental health and psychological wellbeing. None of the 17 surrogates who completed the assessments of psychological health showed signs of depression. Four surrogates scored above the cutoff of 5 on the General Health Questionnaire-30 indicating a 50% likelihood of having a psychiatric condition. Two of the four were in contact with a medical professional about their mental health. The mean score for self-esteem as measured by the Rosenberg Self-esteem Scale was within the normal range. The scores on the Scale of Positive and Negative Experience questionnaire showed moderately positive emotional balance for the majority of participants. Most surrogates scored within the normal range for satisfaction with life and flourishing. Thirteen (62%) surrogates had stayed in contact with the child with 11 describing their relationship as positive.

**LIMITATIONS, REASONS FOR CAUTION:**

Sample size for this study was relatively small and some participants from previous phases were unable to be contacted or declined. Five surrogates had completed 11 surrogacy arrangements between them since Phase 2. Seven of these were gestational arrangements, however, whether donor gametes were used was not recorded.

**WIDER IMPLICATIONS OF THE FINDINGS:**

This is the first study to assess psychological health of surrogates 20 years after the birth of the child. Findings show that the majority of surrogates did not experience psychological problems in the longer term. Future research should focus on understanding what individual and contextual factors contribute to both negative and positive psychological health of surrogates in the longer term.

**STUDY FUNDING/COMPETING INTEREST(S):**

This research was funded by the Wellcome Trust (grant number 208013/Z/17/Z) and by the University of Cambridge’s Returning Carers Scheme. The authors have no conflicts of interest to declare.

**TRIAL REGISTRATION NUMBER:**

N/A.

## Introduction

Despite the growing prevalence of surrogacy globally, and the increasing number of women gestating pregnancies for others, research examining the impact of surrogacy for all those concerned remains sparse. A specific gap in the literature relates to the longer-term impact for surrogates of undertaking surrogacy. While studies have examined the psychological health and experiences of surrogacy for surrogates up to 10 years after the birth of the child (Ciccarelli and Beckman, 2005; Jadva *et al.*, 2015), no studies to our knowledge have examined surrogates’ experiences and psychological health beyond this period.

Most studies of surrogates have focused on their motivations for undertaking surrogacy. Studies within jurisdictions in the global north that permit altruistic or compensated surrogacy arrangements have found that surrogates report wanting to help others as their main motivation (see Kneebone *et al.*, 2022). Surrogates from countries such as India and Iran that permit compensated surrogacy have found surrogates to be primarily motivated by financial reasons and the opportunity to earn money for themselves and their families (Karandikar *et al.*, 2014; Taebi *et al.*, 2020; Kneebone *et al.*, 2022). Only a few studies have examined the psychological impact of surrogacy for surrogates. These studies have shown that while some surrogates may experience psychological difficulties in the weeks following the birth of the child (Parkinson *et al.*, 1999; Jadva *et al.*, 2003), the overall rates of depression have been reported to be relatively low (Söderström-Anttila *et al.*, 2016) and decrease over time. A study of surrogates in India found higher levels of depression among surrogates compared to a comparison group of women expecting their own child, although no differences were found on anxiety or stress (Lamba *et al.*, 2018). This study also found that factors during the pregnancy such as lacking social support, keeping surrogacy hidden from others and receiving criticism for being a surrogate were found to be related to increased levels of depression after the birth of the child (Lamba *et al.*, 2018). Some studies have reported the physical challenges that surrogates may experience, including pregnancy complications (Tehran *et al.*, 2014) and physical pain (Taebi *et al.*, 2020). Variation in how surrogacy is practiced across different cultural contexts including the autonomy and control surrogates have over the treatment would likely impact their experiences.

Studies have reported that positive relationships can be formed between surrogates and intended parents (Papaligoura *et al.*, 2015; Fantus, 2021; Kneebone *et al.*, 2022), which can be long lasting (Blake *et al.*, 2016; Carone *et al.*, 2017; Jadva *et al.*, 2023), though contact has been found to dissipate over time and to vary based on where the surrogate and intended parents are located (Pande 2015; Ziv and Freund-Eschar, 2015; Jadva *et al.*, 2019).

Jadva *et al.* (2003) conducted a study examining the experiences and psychological health of 34 surrogates in the UK who had gestated a surrogacy pregnancy ∼1 year previously. The UK does not permit payment of surrogates, only reasonable expenses, can be given or the amount must be authorized by the family court before a parental order (the legal process through which legal parentage is transferred from the surrogate to the intended parents) can be made. The original study reported that surrogates were primarily motivated by a desire to help a couple become parents. Most of the surrogates (27, 79%) were in contact with the intended parents 1 year after the birth of the child. No differences were found in whether or not traditional or gestational surrogates reported having a special bond with the surrogacy-born child. The only difference found between these two groups was that traditional genetic surrogates were more likely to wish that the child would be told about the surrogacy arrangement. Twenty of these surrogates were revisited 10 years later. Nine of the 12 surrogates who reported feeling a special bond with the child at phase 1 continued to feel a special bond to the child 10 years later. The frequency of contact surrogates maintained with the families lessened over time (Jadva *et al.*, 2015), although the proportion of surrogates who reported positive relationship with the intended parents did not change over the 10-year period. Surrogates were not found to be experiencing psychological problems 1 year or 10 years after the birth of the child. At the 10-year follow-up, in order to increase the sample size, additional surrogates who had given birth to a surrogacy child 5–10 years previously were added to the sample. The 34 surrogates had given birth to 102 babies through surrogacy with a mean per surrogate of three babies. The main motivation for undergoing subsequent arrangements was wanting to help a family have a sibling for an existing child, though wanting to help a childless couple and having a previous positive experience of surrogacy were also reported as common motivating factors.

The current study revisited the surrogates from the last phase of the study, in order to understand the longer-term psychological health outcomes and experiences of surrogates. Qualitative findings about surrogates’ experiences and reflections of surrogacy are presented elsewhere (Shaw *et al.*, 2024).

## Materials and methods

### Participants

The original sample of surrogates at phase 1 of this study was recruited through an ongoing longitudinal study of families created using surrogacy (N = 19) and through the only surrogacy organization in existence at the time called Childlessness Overcome Through Surrogacy (COTS) (n = 15) (Jadva *et al.*, 2003). At phase 2, 10 years after phase 1, 20 of the 34 original surrogates took part. Of the 14 who did not take part at phase 2, 12 could not be contacted and 2 declined to participate. An additional 14 surrogates who had given birth to a surrogacy-born child 5–12 years previously were recruited to the study to increase the sample size (Imrie and Jadva, 2014). The additional participants were recruited through the two UK surrogacy organizations running at that time (Surrogacy UK and COTS) and two UK fertility clinics (Bourn Hall Clinic and CARE Fertility, Manchester). For the current phase of the study, contact was initiated with all 34 surrogates who had taken part at phase 2. Ten participants were uncontactable, one participant initially arranged an interview but did not attend, and two participants declined the invitation to participate due to time constraints. Thus, the present study reports data from 21 surrogates who had completed 71 UK-based surrogacy arrangements (*M *= 3.38; SD = 1.8, Range = 1–6). Twelve surrogates had taken part in phases 1 and 2, and nine surrogates had taken part in phase 2 only and all had carried a pregnancy for heterosexual couples. Ten surrogates (48%) had completed only gestational surrogacy arrangements, five (24%) had completed only traditional surrogacy arrangements, and six (29%) had completed both traditional and gestational surrogacy arrangements. Participants had completed their first successful surrogacy arrangement an average of 20 years ago (*M *= 20.33 years, SD = 3.31, range 13–26 years) for a heterosexual couple. At phase 2, all surrogates had completed arrangements that involved either the intending parents’ gametes or the surrogate’s egg. Five surrogates had completed 11 surrogacy arrangements between them since Phase 2. Of these 11, 7 were gestational surrogacy arrangements; however, whether the intending parents’ gametes or donor gametes were used was not recorded. Comparisons were conducted on measures of depression, relationship quality, and self-esteem at phase 2 between surrogates who took part in phase 3 and those who did not. No differences were found.

Surrogates’ mean age at the time of interview was 52 years (SD = 7.30, range 40–73 years). All surrogates in the current phase had had their own children before they had undertaken their first surrogacy arrangement. Twenty (95%) participants identified as white. One (5%) participant identified as black African/Caribbean. Fourteen (66%) participants worked full or part-time. Most (N = 14, 67%) participants were married to or cohabiting with a partner (see Table 1).

**Table 1.** Demographic information.

	*X*	*SD*
Age of surrogate	*52.38*	*7.11*

	***N***	***%***

Relationship status		
Married	10	48
Cohabiting with partner	5	24
Single	6	28
Surrogate working status		
Full-time	11	52
Not currently working	5	24
Part-time	2	10
Retired	2	10
Part-time student, part-time work	1	5
Occupation		
Managerial/technical	2	10
Skilled non-manual	4	19
Skilled manual	3	14
Partly skilled	7	33
N/A not in work	5	24
No. of surrogacy pregnancies		
1–2	7	34
3–4	7	34
5–6	7	34
Type of surrogacy		
Traditional	5	23.8
Gestational	10	74.6
Both	6	28.6

### Procedure

All surrogates were interviewed online using an in-depth semi-structured interview similar to that used during phase 2 of the study. After the interview, surrogates were asked to complete questionnaires administered electronically to assess psychological health. Ethical approval for this study was obtained from the Cambridge Psychology Research Ethics Committee.

### Measures

#### Mental health

In order to assess levels of depression, participants were asked to complete the Beck Depression Inventory II (BDI-II; Beck and Steer, 1987), a 21-item questionnaire. Scores on the BDI-II range from 0 to 63, with scores between 0 and 13 indicating minimal depression, 14 and 19 indicating mild depression, 20 and 28 indicating moderate depression, and 29 and 63 indicating severe depression. The BDI-II has been reported to show high internal consistency (Cronbach alpha of 0.93 in college students and 0.92 in outpatients) (Beck *et al.*, 1996). The Cronbach’s alpha for the present study was 0.782.

To assess surrogate’s psychiatric symptoms, the General Health Questionnaire (GHQ-30; Goldberg, 1978) was completed by surrogates. The scale is widely used to detect psychiatric disorders in the general population. Scores range from 0 to 30 and value of 5 was used as the threshold value, with a score of 5 or higher indicating a 50% likelihood of the participant having a psychiatric condition.

During the interview, surrogates were asked about their psychological health since their last interview. Specifically, surrogates were asked whether they had sought medical support for any mental health conditions, the severity and duration of any symptoms, and whether they had accessed any therapeutic support or used medication.

#### Psychological wellbeing

The Rosenberg Self-Esteem Scale (RSES) was used to measure surrogates’ levels of global self-esteem (Rosenberg, 1965). The scale comprises 10 items rated on a 4-point Likert scale ranging from 0 strongly agree to 3 strongly disagree. Scores on the scale range from 0 to 30 with higher scores indicating higher levels of self-esteem. Cronbach’s alpha for the present study was 0.89.

Emotional wellbeing was assessed using the Scale of Positive and Negative Experience (SPANE) (Diener *et al.*, 2010). The SPANE is a 12-item questionnaire comprising six items to assess positive feelings (e.g. Good, Joyful) and six items to assess negative feelings (e.g. Angry, sad). Participants are asked to indicate how often they experienced each feeling during the last 4 weeks on a 5-point scale ranging from 1 (very rarely or never) to 5 (very often or always). Scores per subscale are added so that both subscales range from 6 to 30. A balance score is calculated by subtracting the total negative feeling score from the positive feeling score.

The Satisfaction With Life Scale (SWLS) (Diener *et al.*, 1985) was administered to measure global cognitive judgments of satisfaction with one’s life. The scale comprises 5 items rated on a 7-point Likert scale ranging from strongly disagree to strongly agree. Higher scores on the SWLS indicate higher levels of life satisfaction with scores above 30 representing high satisfaction and a score between 5 and 9 indicating extreme dissatisfaction (Arrindell *et al.*, 1991). Cronbach’s alpha for the SWLS for the present study was 0.934.

The Flourishing scale (Diener *et al.*, 2010) was used to assess the surrogates’ self-perceived success in areas such as relationships, self-esteem, purpose, and optimism. The scale has 8 items rated on a 7-point Likert scale ranging from Strongly disagree to strongly agree. Total scores range from 8 to 56 with higher scores representing a person who has many psychological resources and strengths. Cronbach’s alpha for the present study was 0.87.

#### Frequency and type of contact with target parent and child

Data were obtained on the surrogate’s frequency of contact with the mother, the father, and the surrogacy child. This was coded as ‘>1 × week’, ‘1 × week–1 × month’, ‘1 × month–1 × 3 months’, ‘1 or 2 times a year’, or ‘not at all’. The type of contact with members of the surrogacy family was coded into the following categories: letters, phone, text, face-to-face, email, Facebook, or other.

#### Relationship with the target surrogacy family

The surrogate’s relationship with the parents and the child was coded similarly to previous phases using the following categories: ‘negative’, ‘neutral/ambivalent’, or ‘positive’. ‘Negative’ was coded when there was evidence of arguments or a breakdown in communication, ‘neutral/ambivalent’ was coded for a relationship that was described as unproblematic but with a sense of emotional distance, and ‘positive’ was coded when the surrogate described a warm or friendly relationship.

### Data analysis plan

Results are reported descriptively for all variables as inferential statistics were not deemed appropriate given the sample size. For the variables relating to psychological wellbeing, results are reported based on how surrogates scored in relation to norms and cut-offs for the different scales where available. Seventeen of the 21 surrogates completed the questionnaires assessing mental health and psychological wellbeing.

## Results

### Mental health

All the surrogates showed minimal levels of depression according to the BDI-II scale scores with the highest score being 11, i.e. below the threshold for mild levels of depression.

When analysing the results of the GHQ-30, of the 17 surrogates who completed the questionnaire, 4 (23.5%) scored above the threshold of 5 indicating a 50% likelihood of the participant having a psychiatric condition (1 (5.9%) surrogate scored 5, 1 (5.9%) scored 6, 1 (5.9%) surrogate scored 13, and 1 (5.9%) scored 25). The surrogates who scored 5 and 6 had not seen anyone about their mental health. The two surrogates (11.7%) who had scored 13 and 25 had both had contact with either their general practitioner (GP) or seen outpatient services for psychiatric support to deal with issues not directly related to surrogacy.

### Psychological wellbeing

The average score on the RSES was 17.47 (SD = 5.28) which was within the normal range for self-esteem (i.e. between 15 and 25). Five of the 17 (29%) surrogates scored between 10 and 13 indicating low self-esteem. Three (17.6%) scored above the normal range (25+) indicating higher than average self-esteem. The mean scores on SPANE was 23.18 (SD = 3.50) on the positive experience scale indicating moderately high positive emotions and 13.71 (SD = 3.87) on the negative emotions scale indicating moderately low frequency of negative emotions. The average balance score was 9.47 (SD = 6.50) suggesting moderately positive emotional balance.

Scores on the satisfaction with life scale ranged from 6 to 26 with a mean of 14.47 (SD = 5.68). Four (23.5%) surrogates scored below 9 indicating extreme dissatisfaction with life with the remainder scoring within the normal range. The mean scores on the Flourishing Scale were 44.47 (SD = 6.26), with no surrogates scoring below the cutoff of 15, i.e. none showed signs of very low flourishing suggesting possible distress.

### Frequency and type of contact with target parent and child

The frequency of contact and type of contact maintained with the target parent and child can be seen in Table 2. Thirteen (62%) surrogates had stayed in contact with the child, and eight (38%) had not. Of the eight surrogates who were not in contact with the child, two were in contact with the parents and one with the mother only. The surrogate who was not in contact with the child but was in contact with the mother only felt that the level of contact was not enough. In this instance, the child had not been told about the surrogacy as the parents felt the child was not old enough. Of the remaining five, all of whom were not in contact with either parent or the child, four felt that the level of contact was ‘about right’ with one feeling that it was ‘not enough’.

**Table 2.** Frequency and type of contact.

	*N*	*%*
Frequency of contact with child		
>1 × week	2	9.5
1 × week–1 × month	1	4.8
1 × month–1 × 3 months	6	28.6
1 or 2 times a year	4	19.0
Not at all	8	38.1
Total	21	100
Frequency of contact with mother		
>1 × week	2	9.5
1 × week–1 × month	3	14.3
1 × month–1 × 3 months	7	33.3
1 or 2 times a year	3	14.3
Not at all	6	28.6
Total	21	100
Frequency of contact with father		
>1 × week	0	0
1 × week–1 × month	2	9.5
1 × month–1 × 3 months	3	14.3
1 or 2 times a year	7	33.3
Not at all	9	42.9
Total	21	100
Type of contact with child		
Face-to-face	12	57.1
Phone	5	23.8
Text	8	38.1
Facebook	4	19.0
Email	1	4.8
Other (i.e. letters, email, snapchat, Instagram, birthday cards).	5	23.8
Type of contact with mother		
Face-to-face	13	61.9
Phone	13	61.9
Text	15	71.4
Facebook	8	38.1
Email	5	23.8
Other (Instagram).	1	4.8
Type of contact with father		
Face-to-face	12	57.1
Phone	2	9.5
Text	4	19.0
Facebook	3	14.3
Email	0	0
Other	0	0

### Relationship with the surrogacy family

Of the 15 surrogates who remained in contact with the mother, 14 (93.3%) reported a positive relationship with her and one reported a neutral or ambivalent relationship. Of the 12 surrogates who remained in contact with the father, 10 reported a positive relationship, with 1 reporting a neutral or ambivalent relationship and 1 reporting no relationship. Of the 13 surrogates reporting contact with the child, 11 stated a positive relationship and 2 reported that they had no relationship.

## Discussion

This study adds to the growing literature showing that overall, the UK surrogates show good psychological health over the longer term. It further extends what is known already by being the first to report on surrogates who had gestated a surrogacy pregnancy 20 years previously. The findings show that most surrogates did not experience psychological problems in the longer term. Many of the surrogates in the present study reported no problems with their mental health, a finding similar to earlier phases of the study, i.e. at 1 year and 10 years following the birth of the child (Jadva *et al.*, 2003; Imrie and Jadva, 2014).

Unlike previous studies that focused on assessing negative psychological outcomes such as depression and anxiety, the present study included positive aspects of mental health. This decision was taken in part as a result of prior studies showing that surrogates had positive reflections of their involvement in surrogacy. The move to include positive aspects of wellbeing also reflects a wider movement in psychology to move beyond focusing on the absence of mental illness to look at more positive aspects of mental health, which can include flourishing and happiness (Seligman and Csikszentmihalyi, 2000; Seligman, 2011). In relation to psychological wellbeing and life satisfaction for the present study, the findings suggest that surrogates experienced average self-esteem and life satisfaction, relatively high levels of psychological flourishing, and more positive than negative emotions, as reflected in the positive affective balance scores of the SPANE. Thus, overall, in addition to most surrogates not experiencing psychological problems, many experienced positive wellbeing. Indeed, the qualitative analysis of interviews from the present sample showed that many surrogates continued to reflect positively on their experiences of surrogacy with some seeing it as central to their sense of identity (see Shaw *et al.*, 2024). Thus, future research should examine both the negative and positive aspects of psychological well-being to provide a more holistic understanding of mental health for surrogates.

While the majority of surrogates in the present study reported good mental health, some of them did report difficulties and importantly, four did not complete the questionnaire measures. Two surrogates were receiving psychiatric support for issues reported by them to be unrelated to surrogacy. A notable number of surrogates were found to experience low self-esteem (29%) and dissatisfaction with life (23.5%). This finding needs to be interpreted within the context of the age that surrogates were at the time of the study (the mean age of surrogates was 50). Research has found mental health and wellbeing to worsen with age, with midlife being a particularly challenging time (Barbuscia and Comolli, 2021; Gondek *et al.*, 2022). Many arrangements were managed directly between the surrogate and intended parents, which often led to close relationships being formed (Jadva *et al.*, 2003; MacCallum *et al.*, 2003). The present study shows that these relationships can remain positive in the longer term, with over two-thirds of surrogates keeping in contact with the families they had helped 20 years later. In relation to the frequency of contact, for most surrogates, contact with the family ranged from between once or twice a year to monthly. This is similar to a study of surrogates from a single UK fertility clinic which reported 22 of the 30 surrogates to be in contact with the parents more than 8 times a year (Horsey *et al.*, 2022). In the present study, over half of the surrogates met the families in person, however, other means of contact were also utilized with social media contact playing an important role. Social media contact such as Facebook and Instagram allows the different parties to share photographs and feel like a part of each other’s lives with minimal effort and without feeling intrusive. However, for these groups of surrogates, social media was used less often than more traditional means of communication such as phone and text, which may reflect generational differences in modes of communication (Venter, 2017). Importantly, by connecting through social media, the surrogates were able to exchange updates with parents and the child without having a direct relationship with them, and this may explain why some surrogates referred to having no relationship with the parents or child when they were still in touch with each other through social media.

Not all surrogates who had no contact with the family wanted more contact with them, showing the variability in how surrogates can potentially feel about the need for contact with the families they help in this way. It is increasingly common for surrogacy organizations within the UK and overseas to suggest that contact is maintained with the surrogate, however, as was reported in the earlier phase of the present study, this is not always desired by surrogates. Indeed, the relationships may not be close enough to want to maintain contact following the birth of the child, with the earlier phases reporting that wanting to stay in contact was usually a result of forming a close relationship during the pregnancy (Imrie and Jadva, 2014). From the child’s perspective, some surrogacy-born children are curious about their surrogate and may want direct contact with her (Jadva *et al.*, 2023), and thus it may be important for surrogates to maintain a willingness to share information with the child in the future.

The present study has important limitations including the small sample size. Given the long gap between the phases, surrogates may have moved home or changed their contact details, meaning it was not possible to contact all of them. While the analysis of data from phase 2 between those that took part in phase 3 and those that did not, did not show significant differences on the key variables of interest in the study, it is not known if the findings would have been different if all the surrogates who had taken part in the previous phase were included in the current phase. Due to the sample size statistical comparisons could not be conducted between different groups of surrogates as in previous phases of the study, i.e. those who had carried traditional, gestational, or both types of surrogacy, or between those who were previously known to the couple, i.e. a family member or friend, and those who met for the purposes of the surrogacy arrangement. Furthermore, the changes that have taken places in the overall landscape of surrogacy within the UK, for example, increasing numbers of same-sex male couples and single men using surrogacy now compared to 20 years ago, and increasing use of donor gametes, as well as more arrangements being supported by surrogacy organizations and fertility clinics may limit the generalizability of the findings. In the present sample, 5 surrogates had completed 11 surrogacy arrangements between them since Phase 2. Seven of these were gestational arrangements, however, whether donor gametes were used was not recorded. Two-thirds of our sample had completed more than three surrogacy arrangements. The second phase of the study reported the reasons to include wanting to help a family have a sibling for an existing child, wanting to help others or enjoyment of pregnancy (Imrie and Jadva, 2014). It is not known how prevalent repeat surrogacy remains within the UK. Given that surrogacy is now best viewed as an umbrella term (Jadva, 2020), it is important that future research aims to understand the individual and contextual factors that may contribute to both positive and negative outcomes for surrogates.

## Data Availability

The data underlying this article cannot be shared publicly in order to protect the privacy of individuals that participated in the study.
